# Dual RNA Sequencing Reveals the Genome-Wide Expression Profiles During the Compatible and Incompatible Interactions Between *Solanum tuberosum* and *Phytophthora infestans*

**DOI:** 10.3389/fpls.2022.817199

**Published:** 2022-03-03

**Authors:** Honghao Li, Rongping Hu, Zhonghan Fan, Qinghua Chen, Yusong Jiang, Weizao Huang, Xiang Tao

**Affiliations:** ^1^Institute of Plant Protection, Sichuan Academy of Agricultural Sciences, Key Laboratory of Integrated Pest Management on Crops in Southwest, Ministry of Agriculture, Chengdu, China; ^2^College of Life Sciences, Sichuan Normal University, Chengdu, China; ^3^Chengdu Institute of Biology, Chinese Academy of Sciences, Chengdu, China; ^4^Research Institute for Special Plants, Chongqing University of Arts and Sciences, Chongqing, China

**Keywords:** *Phytophthora infestans*, different potato cultivars, compatible interaction, incompatible interaction, dual RNA-seq

## Abstract

Late blight, caused by *Phytophthora infestans* (*P. infestans*), is a devastating plant disease. *P. infestans* genome encodes hundreds of effectors, complicating the interaction between the pathogen and its host and making it difficult to understand the interaction mechanisms. In this study, the late blight-resistant potato cultivar Ziyun No.1 and the susceptible potato cultivar Favorita were infected with *P. infestans* isolate SCPZ16-3-1 to investigate the global expression profiles during the compatible and incompatible interactions using dual RNA sequencing (RNA-seq). Most of the expressed Arg-X-Leu-Arg (RXLR) effector genes were suppressed during the first 24 h of infection, but upregulated after 24 h. Moreover, *P. infestans* induced more specifically expressed genes (SEGs), including RXLR effectors and cell wall-degrading enzymes (CWDEs)-encoding genes, in the compatible interaction. The resistant potato activated a set of biotic stimulus responses and phenylpropanoid biosynthesis SEGs, including kirola-like protein, nucleotide-binding site-leucine-rich repeat (NBS-LRR), disease resistance, and kinase genes. Conversely, the susceptible potato cultivar upregulated more kinase, pathogenesis-related genes than the resistant cultivar. This study is the first study to characterize the compatible and incompatible interactions between *P. infestans* and different potato cultivars and provides the genome-wide expression profiles for RXLR effector, CWDEs, NBS-LRR protein, and kinase-encoding genes.

## Introduction

Late blight, caused by the oomycete *Phytophthora infestans* (*P. infestans*), is a devastating disease in most potato-growing areas worldwide and was responsible for the Irish famine in the mid-19th century. *P. infestans* infects potato and tomato crops at any developmental stage, causing economic losses up to $6 billion annually (Derevnina et al., 2016). Zoospores are the main dispersal forms of *P. infestans* and once they have reached the host surface, the sporangia germinate to produce germ tubes (Boevink et al., 2020). The germ tubes grow on the host surface, forming an appressorium-like swellings or penetrating the anticlinal walls using cell wall-degrading enzymes (CWDEs) upon locating a suitable host entry site (Kubicek et al., 2014; Boevink et al., 2020; Sabbadin et al., 2021).

Potatoes have developed sophisticated surveillance systems, which respond to and prevent pathogenic infections. Among these, the plant cell wall represents the first protective barrier. Moreover, pattern recognition receptors (PRRs) located on the host cell surface detect the evolutionarily conserved pathogen-associated molecular patterns (PAMPs) or apoplastic effector proteins and initiate the PAMP-triggered immunity (PTI) (Dou and Zhou, 2012; Win et al., 2012). In response, pathogens secrete intracellular effector proteins through haustoria to interfere with the PTI and promote their colonization, a phenomenon referred to as effector-triggered susceptibility (ETS) (Whisson et al., 2007; King et al., 2014; Zheng et al., 2014; Boevink et al., 2016; Wang S. et al., 2018). The Arg-X-Leu-Arg (RXLR) is the most studied class of cytoplasmic *P. infectans* effectors, containing a signal peptide followed by the conserved RXLR motif and is associated with the biotrophic phase of *P. infestans* infection (Whisson et al., 2007; Dou et al., 2008; Gilroy et al., 2011; Vleeshouwers et al., 2011; King et al., 2014; Zheng et al., 2014; Boevink et al., 2016). In resistant potato genotypes, effector-triggered immunity (ETI) is induced by direct or indirect recognition of some RXLR effectors by nucleotide-binding site-leucine-rich repeat (NBS-LRR) proteins (R proteins), resulting in localized cell death [hypersensitive response (HR) cell death] (King et al., 2014). The recognized RXLR effectors are referred to as avirulence (Avr) proteins. Several *Avr* genes belonging to the RXLR class of oomycete effectors have been investigated, since the first cloning of *P. infestans Avr* gene (*AVR3a*) in 2005 (Armstrong et al., 2005). These include *AVR2* (Saunders et al., 2012), *AVR3b* (Li et al., 2011), *AVR4* (Van Poppel et al., 2008), *AVRblb1* (Vleeshouwers et al., 2008), *AVRblb2* (Oh et al., 2009), and *AVRvnt1.1* (Pel, 2010). The RXLR effectors are extremely diverse and can rapidly evolve to evade detection by host R proteins (Birch et al., 2008; Raffaele et al., 2010). *P. infestans* genome encodes 563 RXLR effectors (Haas et al., 2009); thus, cultivating resistant potato cultivars is the most effective way of preventing and controlling potato late blight. However, the host-driven selective pressure causes *RXLR* genes to mutate rapidly, enabling *P. infestans* to escape host defense and establish an infection (Yang B. et al., 2017). Since pathogen virulence and host resistance are constantly changing, the evolutionary dynamics of the plant–pathogen interactions can be well illustrated by a four-phased “zig-zag” model (Jones and Dangl, 2006). Although publishing the *P. infestans* and *Solanum tuberosum* (*S. tuberosum*) genomes accelerated the characterization of RXLR effectors (Haas et al., 2009; Xu et al., 2011), most of the 563 RXLR effectors are not yet known. Currently, *P. infestans* effectoromics is employed to identify the potential potato germplasm exhibiting late-blight resistance germplasms. The technique involves transforming and transiently expressing RXLRs recombinant plasmids into plant leaves to determine the existence of potential resistance genes in host materials based on the triggered HR reaction (Vleeshouwers et al., 2008; Oh et al., 2009; He et al., 2019; Ren et al., 2019). While this strategy is reliable, it is greatly but inefficient. Dual RNA sequencing (RNA-seq) is a newly developed method for a comprehensive understanding of the host–pathogen interactions, involving simultaneous analysis of the gene expression changes in both the pathogen and host genomes (Westermann et al., 2012, 2017; Du et al., 2021).

This study employed the dual RNA-seq to investigate the genome-wide expression patterns in two potato cultivars, the late blight-resistant variety (Ziyun No.1) and the susceptible variety (Favorita), infected with *P. infestans* isolate, SCPZ16-3-1. This study was the first study to characterize the compatible and incompatible interactions between *P. infestans* and different potato cultivars, thus providing genome-wide expression profiles for effector, NBS-LRR, and kinase-encoding genes.

## Results

### Interactions Between the Potato Plants and *Phytophthora infestans*

**FIGURE 1:**
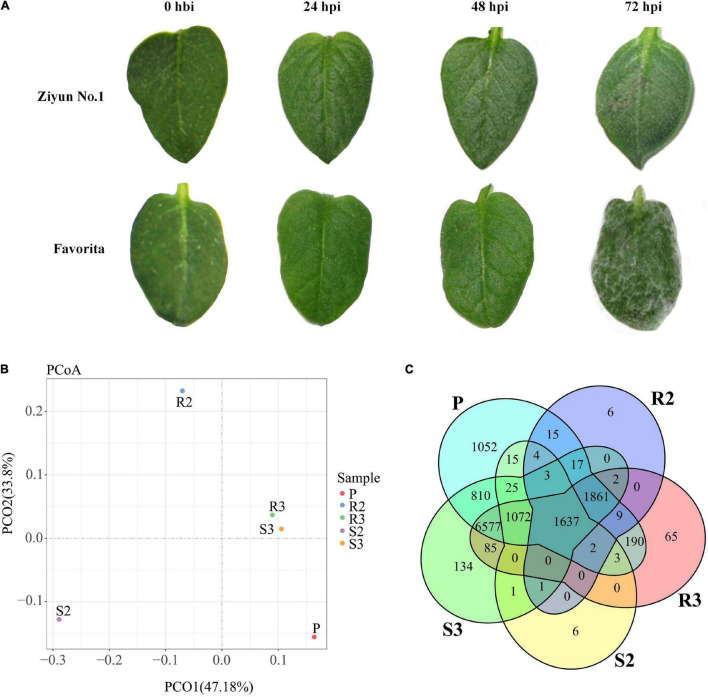
Infection response and global transcriptomic response of *Phytophthora infestans* (*P. infestans*). **(A)** phenotypic responses of *Solanum tuberosum* cv. Favorita and Ziyun No.1 to the infection of *P. infestans*. **(B)** Principal coordinates analysis (PCoA) of *P. infestans* genes. **(C)** Venn diagrams of the *P. infestans* genes expressed in different samples. Hbi, hours before inoculation; hpi, hours post-inoculation. R1, R2, and R3 present samples collected at 0 hbi, 24 hpi, and 48 hpi in incompatible interaction. S1, S2, and S3 present samples collected at 0 hbi, 24 hpi, and 48 hpi in compatible interaction. P presents zoospore sample of *P. infestans*.

Leaves of the 47 potato cultivars were infected *in vitro* with *P. infestans* isolate SCPZ16-3-1 and disease indexes were measured after 5 days (unpublished). Ziyun No.1 had the lowest disease index (5.21 ± 1.19), while Favorita exhibited the highest disease index (100.00 ± 0.29). No late blight lesions were observed on the leaf surface of Ziyun No.1 and Favorita at 24 h post-inoculation (hpi) with the zoospore suspension. However, at 48 hpi, infection spots emerged around the inoculated areas of the Favorita leaves. At 72 hpi, the resistant variety, Ziyun No.1, developed a HR at the site of infection, while Favorita leaf surface was covered with *P. infestans* mycelium (Figure 1A).

### Dual RNA Sequencing of the Potato and *Phytophthora infestans*

To investigate the transcriptomic responses during the interaction between the virus-free potato seedlings and *P. infestans*, we collected Ziyun No.1 and Favorita leaf samples at 0 h before inoculation (hbi) (labeled as R1 and S1), 24 hbi (labeled as R2 and S2), and 48 hbi (labeled as R3 and S3) for the dual RNA-seq analysis. The zoospore suspension was set as the control sample (labeled as P). A total of 339,638,090 clean reads (PE 125 bp) corresponding to 42.76 Gbp were generated by Illumina Hiseq 2000 platform (Table 1). Clean reads of each sample were first mapped independently to the *P. infestans* genome using TopHat2 (Trapnell et al., 2012). At 0 hbi, only 4,133 (R1) and 10,317 (S1) reads, corresponding to a mapping ratio of 0.01 and 0.03%, respectively, were mapped to the reference genome. Thus, these two mapping ratios can be considered false positive, since no zoospore suspension was inoculated at 0 hbi. The ratio of incompatible interaction reached 7.23% (R3), while the ratio of compatible interaction was 14.72% (S3) at 48 hpi, corresponding to 11,503 and 12,225 expressed genes, respectively. The mapping ratio of the zoospore suspension sample was 84.10% and covered 13,292 genes (Table 1). Thereafter, the unmapped reads were retrieved and mapped to the *S. tuberosum* genome (Xu et al., 2011). The mapping ratios of the six samples were 74.05 (R1), 73.80 (R2), 71.72 (R3), 74.70 (S1), 73.39 (S2), and 71.13% (S3), corresponding to 21,859, 22,066, 22,876, 21,916, 22,069, and 22,889 expressed potato genes, respectively.

**TABLE 1:** Comparison statistics of clean reads mapped to *Phytophthora infestans* (*P. infestans*) or genome.

Genotype	Sampling time	Sample name	Total reads	To *P. infestans* genome			To *S. tuberosum* genome		
				Mapped reads	Mapped ratio	Expressed genes	Mapped reads	Mapped ratio	Expressed genes
Ziyun No.1	0 hbi	R1	36,808,120	4,133	0.01%	236	27,254,513	74.05%	21,859
	24 hpi	R2	50,457,514	35,527	0.07%	3,557	37,212,406	73.80%	22,066
	48 hpi	R3	60,736,108	4,391,693	7.23%	11,503	40,411,630	71.72%	22,876
Favorita	0 hbi	S1	38,440,928	10,317	0.03%	338	28,707,398	74.70%	21,916
	24 hpi	S2	66,219,004	37,496	0.06%	2,769	48,568,389	73.39%	22,069
	48 hpi	S3	66,290,776	9,759,740	14.72%	12,225	40,211,904	71.13%	22,889
Zoospore	0 hbi	P	20,685,640	17,396,732	84.10%	13,292			

### Genome-Wide Expression Profiling of the Infection Process

**FIGURE 2:**
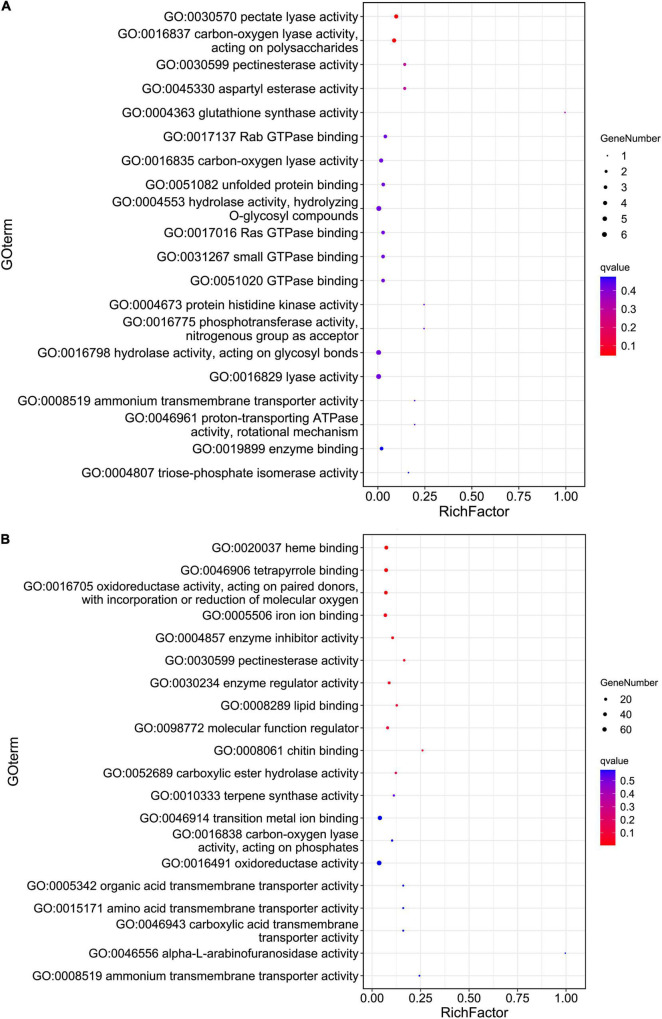
The Gene Ontology (GO) enrichment of the pathogen specifically expressed genes (SEGs) **(A)** and potato SEGs **(B)** identified from compatible interaction. Hypergeometric tests were performed with a threshold value of false discovery rate (FDR) < 0.05 based on the GO classification of 224 pathogen **(A)** and 1,312 potato **(B)** SEGs.

Principal coordinates analysis (PCoA) based on the expression patterns of *P. infestans* genes showed that samples R3 and S3 clustered together, while samples R2, R3, and P dispersed relatively (Figure 1B). A comparison of the expression patterns of *P. infestans* genes showed that only 1,637 genes were expressed consistently across the five samples (R2, R3, S2, S3, and P) (Figure 1C). Moreover, the R2 and S2 samples had six specifically expressed genes (SEGs), while the R3, S3, and P samples had 65, 134, and 1,052 SEGs, respectively (Supplementary Table 1A). The Gene Ontology (GO) enrichment results showed that the 134 SEGs identified in S3 were enriched in pentose and glucuronate interconversions, pectate lyase activity, carbon-oxygen lyase activity, and polysaccharide catabolic and metabolic. Meanwhile, the 1,052 SEGs expressed in the P sample were enriched in pentose and glucuronate interconversions, extracellular region, DNA helicase activity, DNA recombination, telomere maintenance, telomere organization, and anatomical structure homeostasis. Compared with the zoospore sample, the R3 sample had 152 SEGs enriched in the GO terms of the endosomal vesicle fusion. Additionally, the S3 samples had 224 SEGs enriched in pentose and glucuronate interconversions pathway and the GO terms of pectate and carbon-oxygen lyase activities (acting on polysaccharides), compared with the zoospore sample (Figure 2A). The expression patterns between the S samples (S2, S3) and R samples (R2, R3) showed that 285 *P. infestans* gene were specially expressed during the incompatible interactions, while 991 genes were specially expressed during the compatible interactions. The 285 SEGs of the incompatible interaction were enriched in the voltage-gated potassium channel complex and plasma membrane. Conversely, the 991 SEGs of the compatible interaction were enriched in pentose and glucuronate interconversions, betalain biosynthesis pathways, and plasma membrane.

**FIGURE 3:**
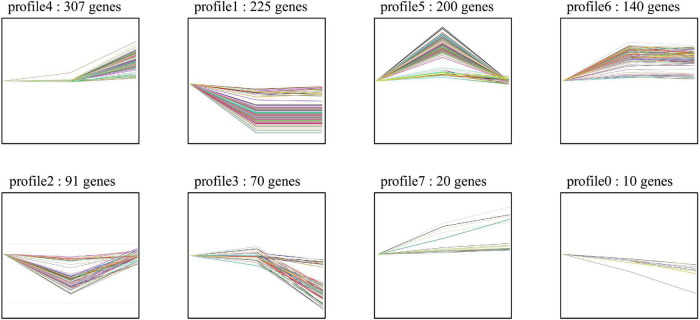
Expression trend of the potato SEGs identified from incompatible interaction. The 1,180 SEGs were identified in Ziyun No.1 when compared with the susceptible cultivar Favorita. Trend analyses were performed based on the expression levels of the 1,180 SEGs at 0 hbi, 24 hpi, and 48 hpi using the OmicShare tools, a free online platform for data analysis (https://www.omicshare.com/tools).

For the host cell, the read mapping ratios decreased with the increase in inoculation time (Table 1). A total of 25,308 *S. tuberosum* genes were expressed in the compatible and incompatible interactions, among which 19,524 genes were consistently expressed in all the six samples (Supplementary Figure 1B and Supplementary Table 1B). Samples R1 and S1 clustered together, while the other samples dispersed relatively, as shown by the PCoA analyses (Supplementary Figure 1A). During the incompatible interactions, 1,180 genes identified as SEGs were enriched in the GO terms of terpene synthase activity, tetrapyrrole binding, carbon-oxygen lyase activity, response to biotic stimulus, and also the phenylpropanoid biosynthesis pathway (Supplementary Table 2A). Eight SEGs involved in response to biotic stimulus all encode kirola-like protein, a member of the SRPBBC (START/RHO_alpha_C/PITP/Bet_v1/CoxG/CalC) ligand-binding domain superfamily. These SEGs included *PGSC0003DMG400002862*, *400007765*, *400007766*, *400007767*, *400042669*, *400013830*, *402009815*, and *400023216*. Besides, five NBS-LRR protein-encoding genes, including *PGSC0003DMG402018953*, *400026469*, *400010886*, *400007872*, and *400002458*, were specially expressed. Expression trend analyses identified 467 upregulated genes from the 1,180 incompatible interaction SEGs (Figure 3), among which 307 genes were upregulated from 24 hbi to 48 hpi, 140 genes were upregulated from 0 hbi to 24 hpi, and 20 genes were upregulated continuously from 0 hbi to 48 hpi. These 467 genes were significantly enriched in biotic stimulus and defense response. Additionally, 1,312 host genes were identified as SEGs during the interaction between *P. infestans* and Favorita (Supplementary Table 2B). They were enriched in the GO terms of tetrapyrrole binding, oxidoreductase, and pectinesterase activities (Figure 2B) and in pentose and glucuronate interconversions, two-component system, and plant–pathogen interaction pathways.

Compared the compatible interaction and incompatible interaction, 338 genes, including 12 *NBS-LRR* genes, 8 disease resistance genes, and 13 kinase genes, were significantly highly expressed in R1, R2, and R3 when compared with S1, S2, and S3, respectively (Supplementary Table 3). A total of 378 genes were highly expressed in R2 and R3 compared with R1 (Supplementary Table 4) and enriched in mitogen-activated protein kinase (MAPK) signaling pathway, plant hormone signal transduction, phenylpropanoid biosynthesis, and stress response. Of the 378 genes, 67 genes were continuously and significantly upregulated during the infection. A total of 581 potato genes exhibited opposite expression trends in the compatible and incompatible interactions, of which 296 genes were upregulated in incompatible interaction and 285 genes were upregulated in compatible interaction (Supplementary Table 5). Out of the 581 genes, 482 genes had their sum of the expression levels lower than 5 Fragments Per Kilobase of exon model per Million mapped (FPKM) (sum < 5 FPKM), while the other 99 genes with sum > 5 FPKM were enriched in the GO terms of response to auxin and stimulus, including several auxin-induced Small auxin-up RNA (SAUR) and NBS-LRR-encoding genes.

Nine *P. infestans* genes (including six RXLR effector-encoding genes) and 11 potato genes (including two NAC transcription factors, four WRKY transcription factors, one MYB transcription factor, and four cytochrome P450-encoding genes) were selected for quantitative reverse transcription PCR (qRT-PCR) verification. Beta-actin gene of potato (*PGSC0003DMG400003985*) and a constitutive expressed gene of *P. infestans* (*PITG_00056*) were used as references (Supplementary Table 6). It was showed that 82.5% qRT-PCR results were consistent (similar variation trend) with RNA-seq (Supplementary Figure 2 and Supplementary Table 6).

### Effector-Encoding Genes of the Pathogen

**FIGURE 4:**
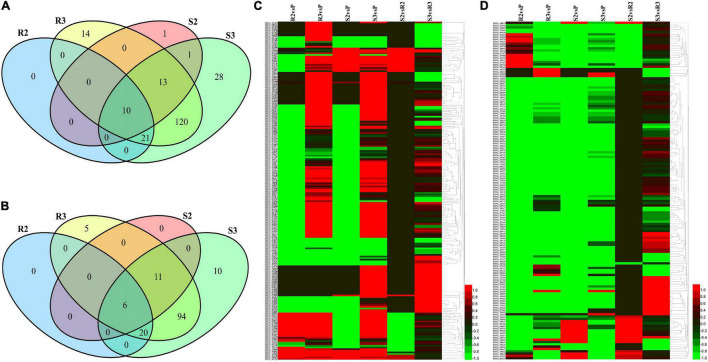
Expression patterns of the effector genes in *P. infestans* during the infection. The log_2_FC values were calculated based on the expression levels and used to draw heatmaps by HemI (version 1.0) (Deng et al., 2014). **(A)** Venn diagram of the 208 Arg-X-Leu-Arg (RXLR) effector genes. **(B)** Venn diagram of the 146 RXLR effector genes. **(C)** Heatmap of the 208 RXLR effector genes. **(D)** Heatmap of the 146 CRN effector genes.

A recently published *P. infestans* genome has been predicted to contain 563 RXLR and 196 Crinkler (CRN) predicted genes (Haas et al., 2009). Except for *PITG_04090*, *15125*, *16427*, *23131*, and *14343* genes, this study identified 559 RXLR effector genes in R2, R3, S2, S3, and P (Supplementary Table 1A). However, only 208 RXLR effectors were expressed during the infection process (Figures 4A,C). Most of the 208 *RXLR* genes showed lower expression levels than the zoospore suspension at 24 hpi, including the 156 and 166 *RXLR* genes downregulated in R2 and S2, respectively. Contrarily, most of the 208 *RXLR* genes were upregulated at 48 hpi (Figure 4A). Further analyses demonstrated that 48 *RXLR* genes were specially but slowly expressed during the infection, mainly at 48 hpi (Figures 4A,C). A total of 195 CRN effector genes were detected in R2, R3, S2, S3, and P samples, but 49 of them were specially expressed in the P sample. Most of them were highly expressed, but a few CRNs were induced during the infection (Supplementary Table 1A and Figures 4B,D).

Seven (*PITG_03192*, *04089*, *04314*, *02860*, *11383*, *22798*, *and 13628*) and nine previously characterized *RXLR* genes (*PITG_03192*, *04089*, *02860*, *09316*, *22798*, *04145*, *06087*, *09585*, and *13628*) were upregulated during the infection in Ziyun No.1 and Favorita, respectively (Table 2). By combining the transcriptome and the published NimbleGen microarray data of *P. infestans* (Haas et al., 2009), we identified 39 upregulated (| log_2_FC| > 1) *RXLR* genes, including seven characterized RXLR effector genes (*PITG_16705*, *13093*, *09218*, *06478*, *00582*, *16195*, and *17316*) and three uncharacterized RXLR effector genes (*PITG_21740*, *23129*, and *13044*) (Table 2).

**TABLE 2:** Upregulated Arg-X-Leu-Arg (*RXLR*) genes identified by microarray (Haas et al., 2009) and RNA sequencing (RNA-seq) during the infection.

GeneID	Gene Name	RNA-Seq expression levels				
		R2	R3	S2	S3	P
*PITG_15110*	−	0.00	36.72	83.28	35.33	16.03
*PITG_21740*	−	0.00	15.11	0.00	18.31	4.47
*PITG_16705*	*AVRcap1b*	581.69	449.92	350.16	478.30	65.86
*PITG_16195*	−	0.00	34.62	0.00	60.55	0.16
*PITG_13093*	−	0.00	279.05	647.64	335.13	30.44
*PITG_06478*	−	0.00	80.49	257.54	87.70	48.80
*PITG_09732*	−	145.80	208.43	263.29	223.81	79.86
*PITG_04196*	*CRE2*	0.00	46.76	0.00	56.12	7.24
*PITG_22804*	−	0.00	182.41	0.00	180.96	29.77
*PITG_10540*	−	0.00	69.04	0.00	85.40	25.60
*PITG_13044*	−	0.00	2.65	0.00	5.75	1.14
*PITG_13048*	−	0.00	197.63	0.00	179.05	68.13
*PITG_00582*	−	0.00	571.82	742.35	574.98	18.81
*PITG_08174*	−	235.16	79.90	0.00	93.74	4.28
*PITG_09216*	−	856.30	2499.00	1030.93	2660.06	660.68
*PITG_10232*	−	0.00	217.37	0.00	218.08	5.89
*PITG_07555*	−	300.01	65.76	0.00	63.29	6.06
*PITG_09160*	*PexRD3*	306.27	594.92	1106.18	595.28	312.47
*PITG_12737*	−	0.00	622.95	0.00	750.66	200.53
*PITG_09218*	−	632.32	1427.03	0.00	1676.08	1027.36
*PITG_05750*	*PexRD49*	0.00	43.66	0.00	59.42	17.15
*PITG_06099*	*PexRD50*	0.00	445.07	590.01	553.32	18.97
*PITG_00774*	−	0.00	39.95	0.00	37.93	0.00
*PITG_22757*	−	0.00	41.62	0.00	67.79	4.42
*PITG_17063*	*PexRD44*	334.14	40.35	0.00	64.44	11.92
*PITG_04049*	−	1383.81	152.49	624.75	174.54	14.62
*PITG_22724*	−	0.00	12.13	0.00	12.65	3.08
*PITG_04266*	−	0.00	148.60	0.00	194.92	12.59
*PITG_10654*	−	0.00	910.64	0.00	1036.31	413.79
*PITG_16294*	*Avr-vnt1*	0.00	110.22	0.00	166.83	44.28
*PITG_21388*	*Avr-blb1*	2940.81	999.01	0.00	1144.18	203.60
*PITG_07594*	−	0.00	22.65	0.00	16.67	4.22
*PITG_22547*	−	0.00	18.88	0.00	13.54	4.73
*PITG_02860*	−	0.00	149.86	0.00	221.55	72.05
*PITG_01934*	−	0.00	132.76	0.00	188.55	1.06
*PITG_14787*	*PexRD2-like-2a*	0.00	0.00	0.03	7.39	0.00
*PITG_06087*	*PexRD16*	534.33	522.77	0.00	680.69	177.90
*PITG_23129*	−	623.57	644.92	0.00	557.29	134.06
*PITG_19942*	−	0.00	83.67	0.00	113.80	14.81

### Cell Wall-Degrading Genes

**FIGURE 5:**
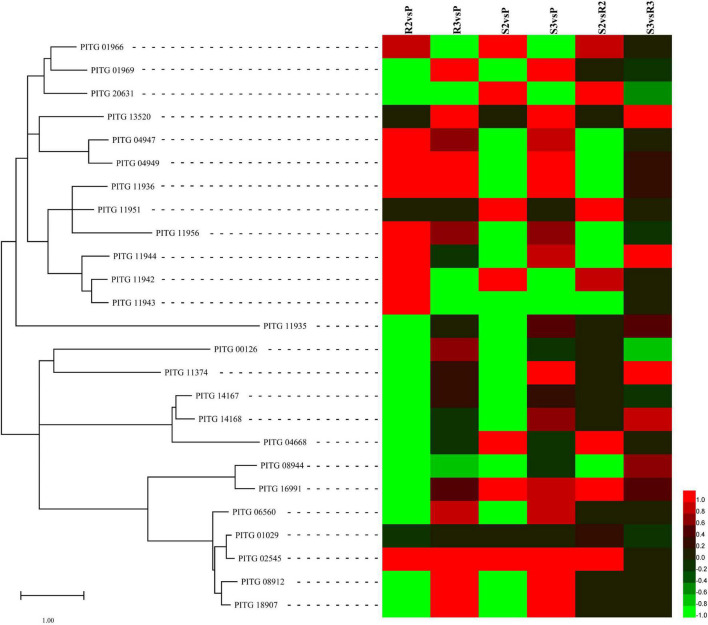
Expression patterns of the differentially expressed cell wall-degrading enzyme (CWDE)-encoding genes. Sequences of CWDEs, including AA17, cellulase, pectin lyases, and pectate lyases, were submitted to MEGA 11 to draw a neighbor-joining tree (Tamura et al., 2021). The log_2_FC values of the *CWDE* genes were calculated based on the expression levels and used to draw heatmaps by HemI (version 1.0) (Deng et al., 2014).

The plant cell wall, which predominantly consists of cellulose and pectin (Burton et al., 2010), represents the first protective barrier against pathogens, promoting plant pathogens to respond by secreting enzymes to depolymerize the main structure of the plant cell wall (Kubicek et al., 2014). A recent study reported that the *P. infestans* genome possesses 31 *AA17* genes encoding copper-bound lytic polysaccharide monooxygenases (LPMOs), which cleave pectin (Sabbadin et al., 2021). In this study, 25 *AA17* genes were expressed (Supplementary Table 7). Most of the *AA17* genes were upregulated, including *PITG_01966*, *01969*, *04947*, *04949*, *11936*, *11942*, *11943*, *11944*, *11951*, *11956*, *13520*, *20631*, and *21641* were upregulated (Figure 5). Among them, *PITG_01966*, *04947*, *04949*, *11936*, *11956*, *18452*, *20312*, and *20631* exhibited the highest expression. For example, the expression levels of *PITG_04947* (*PiAA17A*) were 41.69, 70.19, and 74.20 FPKM in P, R3, and S3 samples, respectively, whereas that for *PITG_04949* (*PiAA17C*) were 65.17, 148.70, and 193.71 FPKM in P, R3, and S3 samples, respectively.

Studies have shown that cellulase (CELB) (EC: 3.2.1.4), pectin lyases (PLs) (EC: 4.2.2.10), and pectate lyases (PELs) (EC: 4.2.2.2) also digest the cell wall (Kubicek et al., 2014). At 48 hpi, three *CELB* genes, *PITG_00126*, *11374*, and *16991*, were upregulated in the compatible and incompatible interactions compared with the zoospore sample (Figure 5). The expression levels of *PITG_00126* were 79.71, 124.71, and 76.42 FPKM in P, R3, and S3 samples, respectively (Supplementary Table 7); however, the gene was not expressed in R2 and S2 samples. The other *CELB* gene, *PITG_16991*, was upregulated at 24 hpi (310.36 FPKM) and 48 hpi (243.49 FPKM) in the compatible interaction. The expression of *PITG_02545*, a PL-encoding gene, was highly induced during the infection and the expression levels were 45.86, 110.46, 188.24, 398.95, and 201.35 FPKM in P, R2, R3, S2, and S3, respectively. Other PL-encoding genes, *PITG_06560*, *08912*, and *18907*, were also induced by the infection of *P. infestans* (Figure 5). However, the expression of the highest expressed *PL* gene, *PITG_01029*, did not change significantly, since its expression levels were 375.67, 331.36, 407.46, 398.95, and 382.37 FPKM in P, R2, R3, S2, and S3, respectively. Another CWDE, PEL, is encoded by *PITG_04668*. During the compatible interaction, *PITG_04668* was significantly induced at 48 hpi. The other two *PEL* genes, *PITG_14167* and *14168*, were relatively highly expressed in P, R3, and S3 samples, with no obvious changes (Figure 5 and Supplementary Table 7).

### Expression Patterns of the Potential Disease-Resistant Genes

**FIGURE 6:**
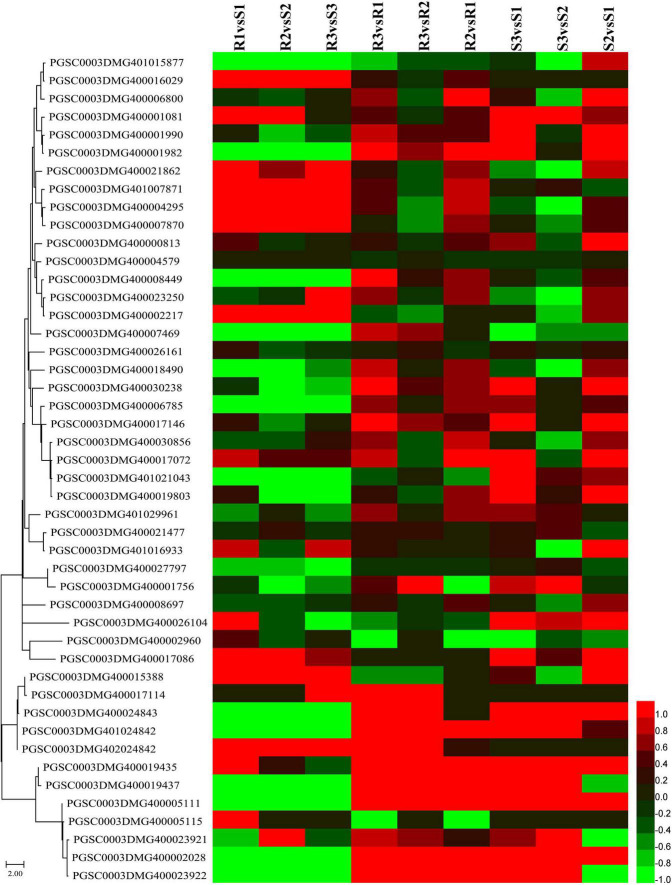
Expression patterns of nucleotide-binding site-leucine-rich repeat (NBS-LRR) and pathogenesis-related protein-encoding genes. Sequences of NBS-LRR and pathogenesis-related proteins were submitted to MEGA 11 to draw a neighbor-joining tree (Tamura et al., 2021). The log_2_FC values of the encoding genes of these proteins were calculated based on the expression levels and used to draw heatmaps by HemI (version 1.0) (Deng et al., 2014). From top to bottom, PGSC0003DMG401015877 to PGSC0003DMG400017086 are *NBS-LRR* genes and PGSC0003DMG400015388 to PGSC0003DMG400023922 are pathogenesis-related protein-encoding genes.

The major late blight resistance genes (*R1*–*R11*) from the wild species *Solanum demissum* were first attracted the attention (Black et al., 1953) and all belong to the NBS-LRR family. This study detected 91 *NBS-LRR* genes, among which 55 genes were slowly expressed (sum of expression levels lower than 5 FPKM) and the other 36 genes were slightly upregulated in R2 and R3 compared to R1 (Figure 6 and Supplementary Table 8). Additionally, several *NBS-LRR* genes, including *402018953*, *400026469*, *400010886*, *400007872*, and *400002458*, were significantly expressed in the incompatible interaction. For example, *PGSC0003DMG400007872* had expression levels 1.22, 1.81, and 1.59 FPKM in R1, R2, and R3, respectively. Twelve *NBS-LRR* genes were highly expressed in the R samples than the S samples (Figure 6 and Supplementary Table 8).

Kinase transfers phosphate groups from high-energy donor molecules (such as ATP) to specific substrates in a process called phosphorylation activating the targets by transmitting upstream signals to the downstream. Studies have reported that several protein kinases also regulate the resistance to late blight in potatoes (King et al., 2014; Ren et al., 2019). We identified 1,197 kinase genes and among these, 212 kinase genes were slowly expressed (the sum of the expression levels in six samples were lower than 5 FPKM) and were excluded from further analysis (Supplementary Figure 3 and Supplementary Table 1B). At 48 hpi, 114 and 84 kinase genes were upregulated, while 45 and five kinase genes were downregulated in R3 compared to R1 and R2. The S1 of Favorita exhibited 299 upregulated (177 and 122) kinase genes at 48 hpi, while 50 and 21 kinase genes were downregulated compared to S1 and S2, respectively, which was higher than in Ziyun No.1. The expression levels of the StMAP3Kε-encoding gene (King et al., 2014), *PGSC0003DMG400007647*, were 24.63, 29.28, 26.85, 21.42, 28.93, and 28.13 FPKM in R1, R2, R3, S1, S2, and S3, respectively. Contrarily, the StMAP3Kβ2-encoding gene (Ren et al., 2019), *PGSC0003DMG400023742*, had expression levels of 25.25, 35.75, 39.34, 24.053, 43.33, and 48.35 FPKM in R1, R2, R3, S1, S2, and S3, respectively. It was found that 83 kinase genes were upregulated and 72 kinase genes were downregulated in S2, while 89 kinase genes were upregulated and 30 kinase genes were downregulated in S3, compared to R2 and R3, respectively.

A total of 13 genes, including three cytoplasmic small heat shock protein class I, two PR1 protein, and two wound-induced protein (WIP), were identified when the keyword “pathogen” was used to search the Non-Redundant Protein Sequence Database (NR), the Kyoto Encyclopedia of Genes and Genomes (KEGG), and the GO annotations (Figure 6 and Supplementary Table 8). Almost all of these genes were significantly upregulated in both the compatible and incompatible interactions with the increasing infection time. The expression levels of *PGSC0003DMG400019435*, a WIP-encoding gene, were 136.81, 329.04, 769.21, 29.85, 275.54, and 1017.35 FPKM in R1, R2, R3, S1, S2, and S3, respectively. An additional WIP gene, *PGSC0003DMG400019437*, was also upregulated at 48 hpi, with the expression levels of 563.67 and 2102.60 FPKM in R3 and S3, respectively.

## Discussion

### Expression Patterns of *Solanum tuberosum* and *Phytophthora infestans* Were Revealed by the Dual RNA Sequencing

*Phytophthora infestans* secretes effector proteins that act in the apoplast or inside living potato cells to interfere with the host immune response, a phenomenon that has contributed to the evolution of a more complex immune system in potatoes (Collinge and Boller, 2001; King et al., 2014; Zheng et al., 2014; Boevink et al., 2016; Yang et al., 2016). However, the prolonged “zigzag” evolution (Jones and Dangl, 2006) of the RXLR effectors, responsible for their extreme diversity, has enabled the pathogen to successfully infect the host plants (Birch et al., 2008; Raffaele et al., 2010). Breeding and cultivating resistant cultivars are the most effective way of preventing and controlling potato late blight; however, the host-driven selective pressure causes the *RXLR* genes to mutate rapidly, allowing *P. infestans* to escape host defense and cause infection (Yang B. et al., 2017). Therefore, understanding the expression profile of RXLR and host genes can pave the way for molecular breeding of disease resistance.

During the compatible interaction with *P. infestans*, 643 (12.9%) differentially expressed genes (DEGs) were identified from 7,680 complementary DNA (cDNA) clones of potatoes (Restrepo et al., 2005). In 2014, a study involving potato inoculation with *P. infestans* identified over 17,000 DEGs, 1,000 secreted proteins, and 40 putative effector targets and reported that the compatible and incompatible interactions exhibited opposing expression patterns of the 50 differentially expressed proteins (Ali et al., 2014). Additionally, Duan et al. identified 8,881 and 7,209 DEGs when *Stagonosporopsis andigena* interacted with *P. infestans* isolate 90128 (incompatible) and CN152 (compatible), respectively (Duan et al., 2020). This study found that the salicylic acid-, jasmonic acid-, and abscisic acid-mediated signaling pathways were activated in response to *P. infestans* infection, while ethylene- and brassinosteroids-mediated defense pathways were suppressed (Duan et al., 2020). However, the transcriptomic responses by *S. tuberosum* to *P. infestans* infection remain largely unknown. Dual RNA-seq shows major advances in robustness, resolution, and interlab portability compared to probe- or tag-based methods (AC’t Hoen et al., 2008) and is a preferred method for studying pathogen–host interactions (Westermann et al., 2012). Frades et al. (2015) developed a novel workflow for identifying families with an expanded or depleted number of transcripts based on the *P. infestans* resistance level by comparing the *de novo* assembled RNA-seq reads of wild *Solanum* species with *P. infestans*. However, the *de novo* assembly of RNA-seq reads from different species results in various misassembles. The publication of *S. tuberosum* (Xu et al., 2011) and *P. infestans* T30-4 (Haas et al., 2009) genome have facilitated the characterization of RXLR effectors and host R genes. In this study, the genome of *S. tuberosum* and *P. infestans* served as references for evaluating the compatible and incompatible interactions between potato and *P. infestans* using dual RNA-seq. Nine members of the 40 putative effector targets, identified by Ali et al. (2014), were downregulated in the interaction between *P. infestans* and Favorita. They included *PGSC0003DMG400002590*, *400009921*, *400010225*, *400012089*, *400014590*, *400028238*, *400028627*, *400028674*, and *401000527*. Besides, 99 protein-encoding genes showed contrasting expression trends (| log_2_FC| > 1.0) in the compatible and incompatible interactions, but did not overlap with the genes identified by Ali et al. (2014).

### Various Biotic Stress-Responsive Genes Were Stimulated

Pathogens secrete intracellular effector proteins into host cells and interfere with PTI to promote colonization (Whisson et al., 2007; King et al., 2014; Zheng et al., 2014; Boevink et al., 2016; Wang S. et al., 2018). When cultivars Ziyun No.1 and Favorita were infected with *P. infestans* isolate SCPZ16-3-1, only 208 RXLR and 146 CRN effector genes were expressed, suggesting that this isolate selectively expressed a part of RXLR effector genes. Thus, different isolates should have different RXLR expression patterns. The necessity to pass through the plant cell wall is another important aspect of pathogenic infections. A recent study reported that 31 copper-bound LPMOs (also named *AA17* genes), which oxidatively cleave the pectin backbone to drive plant infection, are coded by the *P. infestans* genome (Sabbadin et al., 2021). We identified 25 members of the 31 *AA17* genes in at least one sample. Consistent with the results by Sabbadin et al. (2021), *PITG_01966*, *04947*, *04949*, *20312*, and *20631* were highly expressed during the infection and 13 *AA17* genes were upregulated, including ten (*PITG_01966*, *01969*, *04947, 04949*, *11936*, *11942*, *11944*, *11951*, *11956*, and *13520*) of the 11 upregulated *AA17* genes identified by Sabbadin et al. (2021). These results confirmed that *P. infestans* secrete pectin monooxygenases to drive potato infection and validated the reliability of our transcriptome quantitative results. Functional enrichment analysis can determine the overrepresented class of genes, which may be associated with disease phenotypes. When the late blight susceptible cultivar Favorita was infected with *P. infestans*, the SEGs were enriched in pectate and carbon-oxygen lyase activities (acting on polysaccharides) and polysaccharide metabolic process, involved in plant cell wall degradation. The susceptible host activated the genes involved in plant–pathogen interaction pathways in response. *P. infestans* altered the expression of CWDEs to digest the cell wall of the susceptible host (Sabbadin et al., 2021), generating more cell wall degradation products, which affect the structure and gene expression patterns of the host cell. The potato SEGs identified in the incompatible interaction were significantly enriched in phenylpropanoid biosynthesis pathway, biotic stimulus, and defense response, all involved in abiotic stress responses.

Remarkable progress has been made in the pathology of late blight of potato since the first *P. infestans Avr* gene (*AVR3a*) was cloned in 2005 (Armstrong et al., 2005). Over 20 late blight resistance genes, all belonging to the NBS-LRR family, have been cloned and characterized (Ballvora et al., 2002; Song et al., 2003; Van Der Vossen et al., 2003; Huang et al., 2005; Vleeshouwers et al., 2008; Foster et al., 2009; Lokossou et al., 2009; Pel et al., 2009; Stefańczyk et al., 2017, 2020; Yang L. et al., 2017). The upregulation or special expression of many *NBS-LRR* genes in cultivar Ziyun No.1 in this study may indicate its resistance to *P. infestans* isolate SCPZ16-3-1. Kinase-catalyzed phosphorylation activates the target protein and transmits the signals to its downstream. Many protein kinases are involved in the immune signal transduction pathways and play important roles in potato resistance to *P. infestans*. They include receptor-like kinases, such as *StLRPK1* (Wang H. et al., 2018), and mitogen-activated protein kinase kinase kinases, such as *StMAP3K*β*2* (Ren et al., 2019) and *StMAP3K*ε (King et al., 2014). The expression patterns of the kinase genes showed that the compatible interactions elicited more responses than the incompatible interactions. Although pathogen-related proteins play a major role in plant defense and general stress responses, most *NBS-LRR* genes were slowly expressed in Ziyun No.1 and Favorita. However, more than half of the pathogenesis-related genes were highly expressed, especially the basic pathogenesis-related protein 1 (*PGSC0003DMG400005111*) and wound-induced protein (*PGSC0003DMG400019435*, *400019437*). The significant upregulation (> 1,000 FPKM) of wound-induced protein might suggest its important role during the infection even though the protein was not characterized.

## Conclusion

This study employed a timecourse dual RNA-seq to evaluate the compatible and incompatible interactions between *P. infestans* isolate SCPZ16-3-1 and two different potato cultivars. *P. infestans* induced more pathogenesis-related genes, including RXLR effectors and CWDEs-encoding genes, to promote colonization during the compatible interaction. Moreover, the resistant potato activated a set of biotic stimulus responses and the phenylpropanoid biosynthesis genes, while the susceptible potato cultivar upregulated more kinase and pathogenesis-related genes.

## Materials and Methods

### Plant Materials

Virus-free seedlings were obtained from two potato cultivars, late blight-resistant cultivar (Ziyun No.1) and the susceptible cultivar (Favorita) and cultivated on Murashige and Skoog (MS) medium (7%) for 28 days in a growth chamber under a 16/8 h day/night photoperiod, with a light intensity of 2,000 lux, day/night temperature of 25/22°C, and relative humidity of 70%. Thereafter, the seedlings were transferred into plastic pots filled with TS1 fine matrix (Klasmann-Deilmann, Germany) for 10–15 days. After reaching the 6-leaf stage, 20 healthy plants were selected from each cultivar and four leaves of each plant sprayed with 10 μl of the encysted zoospore suspension from *P. infestans* isolate SCPZ16-3-1 (5 × 10^4^ sporangia/ml). The zoospores of this isolate can germinate in 2 to 3 h, fully infect in 24 h, and spread throughout the host leaf in 48 h. The plants were then incubated in darkness for 12 h followed by a 16/8 h day/night photoperiod. Each plant was covered by a clear plastic cup. Leaves were sampled at 0 hbi and at 24 and 48 hpi and immediately snap-frozen and stored in liquid nitrogen. For each sample, ten infected leaves were pooled from five plants. The six samples, including three incompatible interaction samples (R1, R2, and R3) and three compatible interaction samples (S1, S2, and S3), were collected for the following analysis.

### Ribonucleic Acid Isolation, cDNA Library Preparation, and Illumina Sequencing

Total RNA was extracted from leaf samples using TRIzol^®^ reagent (Invitrogen, United States) following the manufacturer’s instructions and DNase I treatment was used to remove the genomic DNA (Fermentas, United States). An Agilent 2100 Bioanalyzer was used to measure the RNA integrity number (RIN), while the RNA quality and purity were assessed using a nanodrop at the ratios of A260/A280 and A260/A230. The RNA samples that met all the quality standards (RNA concentration ≥ 100 ng/μl, A260/A280 ≥ 1.8, A260/A230 ≥ 1.8, RIN ≥ 8, and 28S/18S > 1) were then subjected to cDNA library construction at the Biomarker Technologies Corporation (Beijing, China). Qualified libraries were submitted for transcriptome sequencing at the Illumina Hiseq 2000 platform and paired-end (PE) read sequences were obtained through image analysis and base calling of the sequencing data. The adapter sequences and low-quality reads were eliminated from raw data and the clean PE reads (PE 125 bp) were deposited in the Sequence Read Archive (SRA) database^1^ under the accession numbers of SRR12357394 to SRR12357399.

### Reads Mapping and Sequence Annotation

The genome data of *S. tuberosum*^2^(Xu et al., 2011) and *P. infestans* T30-4^3^(Haas et al., 2009) were downloaded from the National Center for Biotechnology Information (NCBI) database. Clean PE reads obtained from each leaf sample were mapped independently to the *P. infestans* genome (used as a reference) using TopHat2 (Trapnell et al., 2012) and the unmapped reads were then mapped to the *S. tuberosum* genome. Thereafter, aligned reads were assembled using Cufflinks (Trapnell et al., 2012) and merged with the reference annotation using Cuffmerge (Trapnell et al., 2012).

### Expression Pattern Analyses

The mapped read count of each gene and transcript was normalized and quantified using Cuffdiff [1]. Subsequently, pairwise comparisons were performed and false discovery rates (FDRs) were estimated using the theoretical *p*-values and Benjamini–Hochberg method (Benjamini and Hochberg, 1995). The DEGs with a threshold of | log_2_ fold-change| (| log_2_FC|) ≥ 1 and FDR < 0.05 were considered significant. Additionally, hypergeometric test with a threshold value of FDR < 0.05 was used for the GO and the KEGG enrichment analyses.

### Real-Time Quantitative Reverse Transcription-PCR

Genes with relatively higher expression levels were selected for primer design (Additional file 7: Supplementary Table 6) and real-time qRT-PCR analysis. Subsequently, the first strand of cDNA was synthesized using the TransScript First-Strand cDNA Synthesis Kit (AiDLAB Biotech, PR China) according to the manufacturer’s instructions. Real-time qRT-PCR was performed on the AnalytikJena-qTOWER 2.2 Thermocycler (Jena, Germany) using SYBR Green QPCR Mix (DF Biotechnology, China). The reaction mixture included 5 μl 2 × SYBR^®^ Green Supermix, 0.5 μl forward primer, 0.5 μl reverse primer, 1 μl cDNA, and 3 μl ddH_2_O. Moreover, the amplification conditions were: initial denaturation at 95°C for 3 min, followed by 45 cycles of denaturation at 95°C for 10 s, annealing at 60°C for 30 s, and extension at 72°C for 30s. Three biological replicates were used for the amplification and the amplification specificity of the amplicons was determined through the melt curve analysis at 60–95°C. The expression levels of potato genes were quantized for samples R1, R2, and R3, whereas the expression levels of *P. infestans* genes were quantized for samples R2, R3, and P. The relative quantification results were then calculated using the delta-delta Ct (2^–ΔΔCT^) method (Livak and Schmittgen, 2001; Supplementary Table 6).

## Data Availability Statement

The original contributions presented in the study are publicly available. This data can be found here: National Center for Biotechnology Information (NCBI) BioProject database under accession number PRJNA646588.

## Author Contributions

HL, XT, and WH: conceptualization and writing-original draft preparation. XT and YJ: formal analysis. HL and XT: investigation and funding acquisition. HL, RH, ZF, QC, XT, and WH: writing-review and editing. All authors have read and agreed to the published version of the manuscript.

## Conflict of Interest

The authors declare that the research was conducted in the absence of any commercial or financial relationships that could be construed as a potential conflict of interest.

## Publisher’s Note

All claims expressed in this article are solely those of the authors and do not necessarily represent those of their affiliated organizations, or those of the publisher, the editors and the reviewers. Any product that may be evaluated in this article, or claim that may be made by its manufacturer, is not guaranteed or endorsed by the publisher.

## Footnotes

1
http://www.ncbi.nlm.nih.gov/Traces/sra/sra.cgi?

2
https://www.ncbi.nlm.nih.gov/assembly/GCA_000226075.1/

3
https://www.ncbi.nlm.nih.gov/assembly/GCA_000142945

## Supplementary Material

Supplementary Figure 1 **(A)** Principal coordinates analysis (PCoA) of *S. tuberosum* genes; **(B)** venn diagrams of the *S. tuberosum* genes expressed in different samples.

Supplementary Figure 2 Quantitative reverse transcription-PCR (qRT-PCR) verification of RNA sequencing (RNA-seq) data.

Supplementary Figure 3 Expression patterns of kinase-encoding genes.
