# Supplementary figures and images for: Dual RNA Sequencing Reveals the Genome-Wide Expression Profiles During the Compatible and Incompatible Interactions Between Solanum tuberosum and Phytophthora infestans
Source: Front Plant Sci. 2022 Mar 3;13:817199. doi: 10.3389/fpls.2022.817199 (PMC8993506; doi:10.3389/fpls.2022.817199)

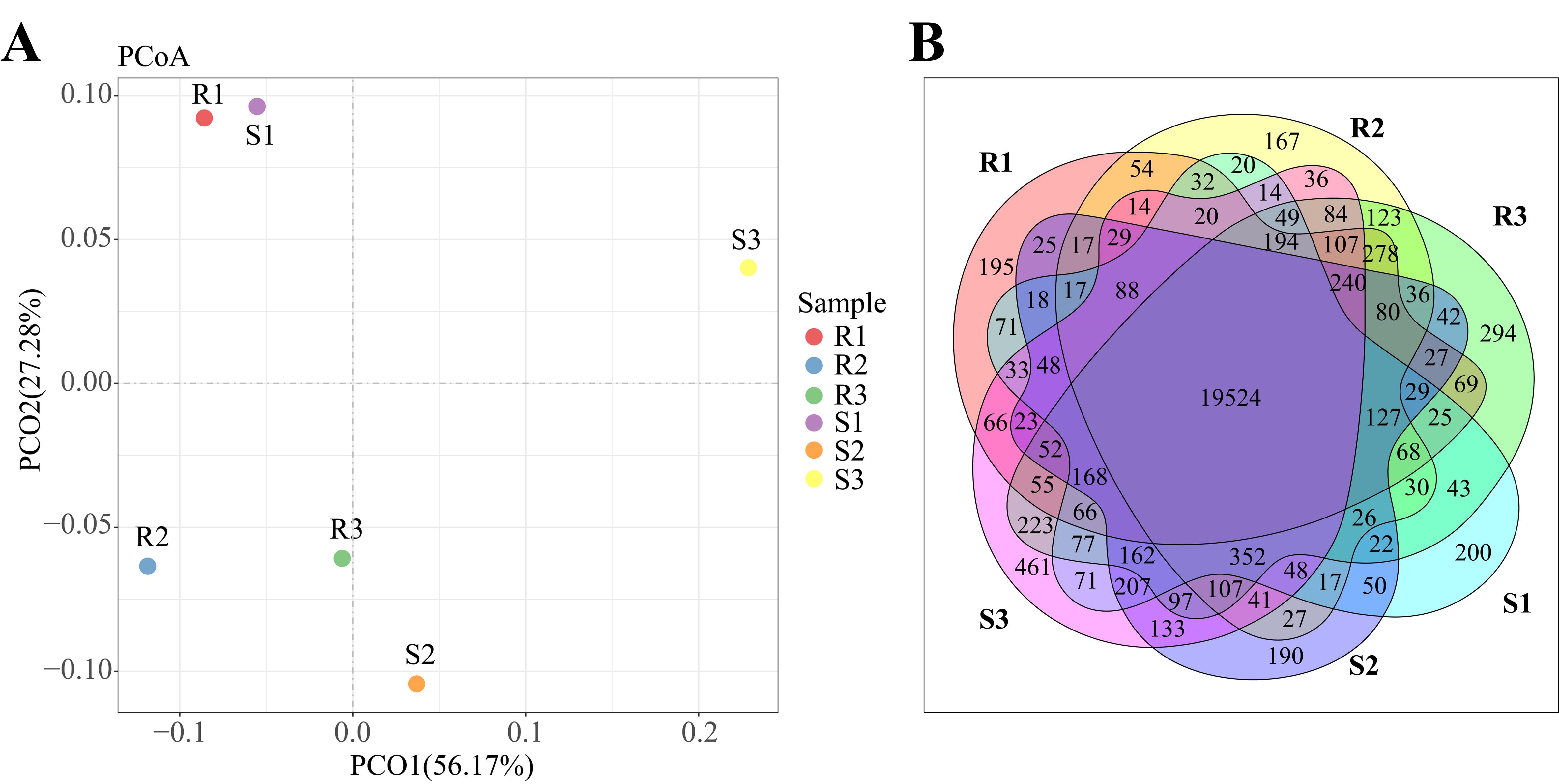

Supplement: Supplementary Figure 1 — (A) Principal coordinates analysis (PCoA) of S. tuberosum genes; (B) venn diagrams of the S. tuberosum genes expressed in different samples. [file Image_1.JPEG]

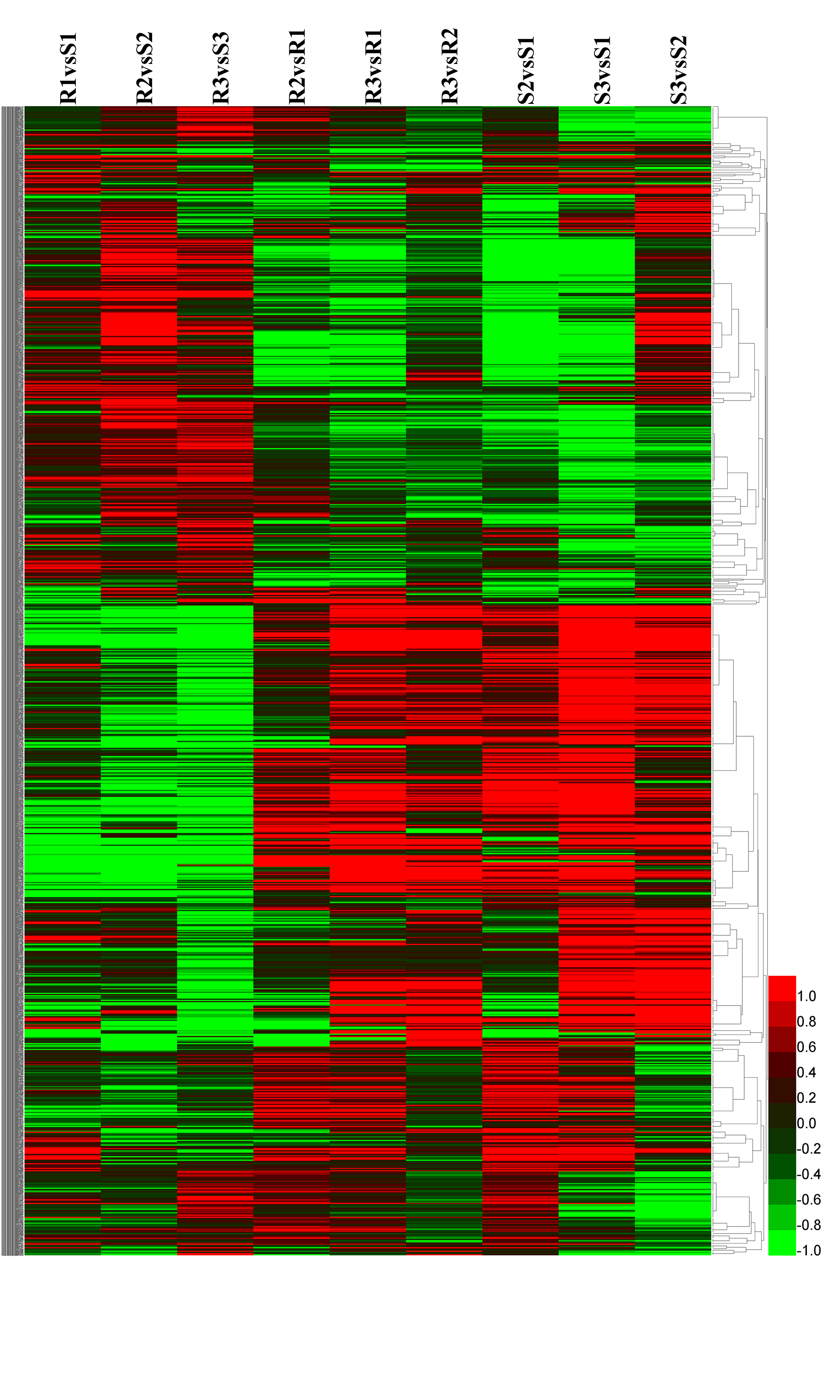

Supplement: Supplementary Figure 2 — Quantitative reverse transcription-PCR (qRT-PCR) verification of RNA sequencing (RNA-seq) data. [file Image_2.TIFF]

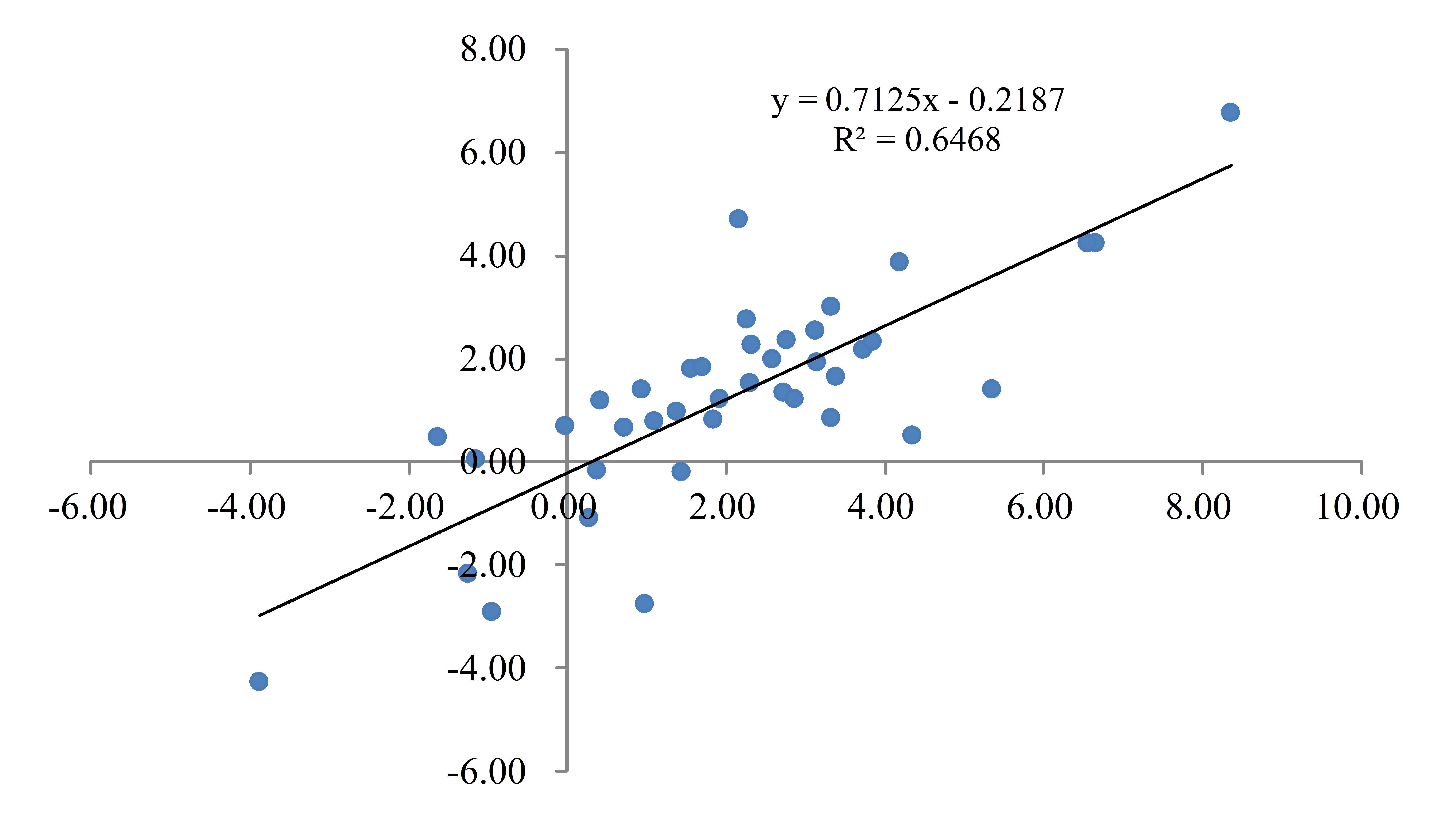

Supplement: Supplementary Figure 3 — Expression patterns of kinase-encoding genes. [file Image_3.JPEG]
